# The Novel *Agrotis ipsilon* Nora Virus Confers Deleterious Effects to the Fitness of *Spodoptera frugiperda* (Lepidoptera: Noctuidae)

**DOI:** 10.3389/fmicb.2021.727202

**Published:** 2021-11-15

**Authors:** Tong Li, Ruobing Guan, Yuqing Wu, Su Chen, Guohui Yuan, Xuexia Miao, Haichao Li

**Affiliations:** ^1^Institute of Plant Protection/Henan Key Laboratory of Crop Pest Control/Key Laboratory of Integrated Pest Management on Crops in Southern Region of North China, Henan Academy of Agricultural Sciences, Zhengzhou, China; ^2^State Key Laboratory of Wheat and Maize Crop Science/College of Plant Protection, Henan Agricultural University, Zhengzhou, China; ^3^Key Laboratory of Insect Developmental and Evolutionary Biology, CAS Center for Excellence in Molecular Plant Sciences, Shanghai Institute of Plant Physiology and Ecology, Innovation Academy for Seed Design, Chinese Academy of Sciences, Shanghai, China

**Keywords:** *Agrotis ipsilon*, *Spodoptera frugiperda*, Nora virus, virus genome, fitness

## Abstract

In the present study, we identified a novel, positive-sense single-stranded RNA virus in the Chinese black cutworm, *Agrotis ipsilon*. It has a genome length of 11,312 nucleotides, excluding the poly(A) tails, and contains five open reading frames. The ORF2 encodes the conserved domains of RNA helicase and RNA-dependent RNA polymerase, while ORF4 and 5 encode three viral proteins. Herein, the *A. ipsilon* virus was clustered with a *Helicoverpa armigera* Nora virus and was thus provisionally named “*Agrotis ipsilon* Nora virus” (AINV). AINV was successfully transmitted into a novel host, *Spodoptera frugiperda*, through injection, causing a stable infection. This found the possibility of horizontal AINV transmission among moths belonging to the same taxonomic family. Nonetheless, AINV infection was deleterious to *S. frugiperda* and mainly mediated by antiviral and amino acid metabolism-related pathways. Furthermore, the infection significantly increased the *S. frugiperda* larval period but significantly reduced its moth eclosion rate. It suggests that AINV is probably to be a parasitic virus of *S. frugiperda*.

## Introduction

The second-generation sequencing technology has expanded our understanding of the diversity of insect viruses (Xu et al., 2014; Liu et al., 2017; Shi et al., 2018; Yang et al., 2019). Recently, picorna-like symbiotic viruses are commonly identified in insects (Shi et al., 2016; Yang et al., 2016; Cholleti et al., 2018). Taxonomically, picorna-like viruses belong to the order *Picornavirales*, which share some properties with members of the family *Picornaviridae* (Le Gall et al., 2008). Generally, picorna-like viruses possess a (+) ssRNA genome (positive-sense, single-stranded RNA), and their translated polyproteins are cleaved into structural and non-structural proteins. Their pathogenicity is diverse, ranging from lethal to symbiotic (de Miranda et al., 2010; Jakubowska et al., 2014).

The Nora virus was first reported in *Drosophila melanogaster* and then proposed to represent a new picorna-like virus family (Habayeb et al., 2006). Subsequent studies have revealed that Nora viruses interfere with the *D. melanogaster* RNAi system (van Mierlo et al., 2012, 2014). Recently, additional members of the Nora virus clade have been identified in arthropods through high-throughput sequencing technologies (Shi et al., 2016). *Agrotis ipsilon* (Hufnagel) is a worldwide pest that causes significant damage to vegetables and grains. Despite its interactions with its endosymbionts being thoroughly studied (Shi et al., 2013), its viral landscape remains ambiguous. Herein, we identified a novel Nora virus in Chinese *A. ipsilon* through RNA sequencing. The virus was provisionally named as “*Agrotis ipsilon* Nora virus” (AINV). The complete AINV genome was subsequently determined, and its phylogenetic position and gene expression patterns were uncovered among the *A. ipsilon* developmental stages. AINV was also introduced into wild *Spodoptera frugiperda*, an invasive pest in China (Sun et al., 2021), to evaluate its effects on fitness and gene expressions.

## Materials and Methods

### Insect Rearing

Colonies of *A. ipsilon* and *S. frugiperda* were established with caterpillars collected in Shanghai, China, in 2018 and Zhengzhou, China, in 2020, respectively. The caterpillars were fed with artificial diets at 25°C (±1°C) under a 14/10h light/dark cycle. Adult moths were fed with 10% honey water.

### Virus Detection in *A. ipsilon* Using RNA-Seq

Total RNA was extracted from *A. ipsilon* samples using the TRIzol reagent (Invitrogen, Carlsbad, CA, United States) following the manufacturer’s protocol. The samples were collected at four developmental stages, i.e., egg and larvae (*n*=30), pupae (*n*=30), and adults (*n*=30) in both male and female insects. RNA quality and quantity were assessed on an Agilent 2,100 Bioanalyzer (Agilent Technologies, Palo Alto, CA, United States) and RNase-free agarose gel electrophoresis. The mRNA in the RNA samples was enriched by removing rRNA using the Ribo-ZeroTM Magnetic Kit (Epicentre, Madison, WI, United States). The mRNA libraries were subsequently prepared by BGI (Shenzhen, China) and sequenced on an Illumina Hiseq 2000 platform. *De novo* transcriptome assemblies were performed using the SOAPdenovo software with default parameters (Xie et al., 2014). Protein sequences of sRNA viruses (txid 439,488), dsRNA viruses (txid 35,325), and Delta viruses (txid 39,759) were retrieved from the NCBI RefSeq database (Reference Sequences) and employed as subjects in local blastx searches to uncover possible viral fragments in assembled unigenes. The non-virus originating hits were filtered by performing online blastx searches against the NCBI nr database (non-redundant protein sequences). An e-value threshold of 1×10^−5^ was used in these searches.

### AINV Genome Amplification and Phylogenetic Analysis

Full-length cDNA sequences of the AINV were obtained using the 3′ and 5′ rapid amplification of cDNA ends (RACE) system (Life Technologies, Carlsbad, CA, United States), following the manufacturer’s instructions. The sequences were subsequently verified through amplification and sequencing using specific primers. Total RNA for specific RT-PCR was extracted using the TaKaRa MiniBEST Universal RNA Extraction Kit (Takara, Dalian, China), followed by RT-PCR amplification of the AINV fragments using PrimeScript^™^ One-Step RT-PCR Kit Ver.2 (Dye Plus; Takara). The gene-specific primers used herein were listed in Table 1. The standard genetic code was employed to predict the open reading frames of the AINV using the NCBI online ORF finder program.^1^ Conserved domains within the ORFs were subsequently predicted using the NCBI conserved domain database v3.17,^2^ with threshold of 1×10^−2^.

**Table 1:** Primers used in this study.

Primer name	Primer sequence (5'-3')	Introduction
AINV-F-1	CTTCTACTCCCAGTGAGTAC	Amplification of AINV Genome
AINV-F-2	GAGGTAGCATACTGCGATG	Amplification of AINV Genome
AINV-R-1	GATTCTTACGCAAGTGACG	Amplification of AINV Genome
AINV-F-3	CTGTGCAAGCGCAGTTGAATC	Amplification of AINV Genome
AINV-R-2	GATATCGTCGACGGGTGGTGTGG	Amplification of AINV Genome
AINV-F-4	GTTGACAGCCGCTATCGTTG	Amplification of AINV Genome
AINV-R-3	GTGATTGGGCATGAGCGCCTCT	Amplification of AINV Genome
AINV-F-5	ATCAGGCTGCAAAGCCTGTG	Amplification of AINV Genome
AINV-R-4	CGAAGCAACCAACACTACAC	Amplification of AINV Genome
AINV-F-6	CTAGCTCTGATCGTCAAC	Amplification of AINV Genome
AINV-R-5	GCCTCGAATACCGTAAGG	Amplification of AINV Genome
AINV-F-7	GAGTCTGGAGAACGGTTCTC	Amplification of AINV Genome
AINV-R-6	CGGATGCTCTATAACGCAC	Amplification of AINV Genome
AINV-F-8	GCGATCTAATCGGACCACGAAC	Amplification of AINV Genome
AINV-R-7	CCTGATGTGTCTGTCATGGTG	Amplification of AINV Genome
AINV-F-9	CAACGTCACTATGTCAGGTC	Amplification of AINV Genome
AINV-R-8	GATCGCGGATGTCGCAATATCC	Amplification of AINV Genome
AINV-F-10	GCAGCATCCATTGCACATG	Amplification of AINV Genome
AINV-R-9	CACAAAACGTTTTCAAG	Amplification of AINV Genome
RACE-3-F	CCGTTTCACAGGAGCGGAGTAATG	RACE
RACE-5-R	CGATCGCTAAATCGGATTTCTCC	RACE
AINV_72F	TGACCGGTACCGATTTTCTC	AINV detection
AINV_44R	CCTGGTATCGACCGTCTGTT	AINV detection
SFACTINF	TGTCTCCCACACCGTCCCCAT	Quantification of *S. frugiperda* β-actin gene
SFACTINR	ACGAACGATTTCCCTCTCAGC	Quantification of *S. frugiperda* β-actin gene
AINVQF	CCAATCACCGTAACCTTA	Quantification of AINV
AINVQR2	ACAACCATAACTGCTGAA	Quantification of AINV

The RNA-dependent RNA polymerase (RdRp) protein sequences from taxa within *Picornavirales* and *Caliciviridae* were retrieved to uncover the phylogenetic position of AINV. The sequences were aligned using the MUSCLE program in MEGA 7.0 (Kumar et al., 2016) and then trimmed using trimAl to remove the poorly aligned regions (Capella-Gutierrez et al., 2009). Phylogenetic analysis was performed in IQ-TREE 1.6.6 (Nguyen et al., 2015), followed by resampling 1,000 ultrafast bootstraps to assess the support for each node. The substitution model based on the Bayesian information criterion in ModelFinder (Kalyaanamoorthy et al., 2017) was selected to improve the accuracy of phylogenetic estimates.

### AINV Transmission and Quantification in a Novel Host

An AINV infected liquid was formulated following the methods described by Xu et al. (2020). The AINV infected fourth *A. ipsilon* larvae were ground in liquid nitrogen and then homogenized with 1ml PBS buffer (0.01M, pH 7.4). The homogenate was then centrifuged at 6500×g for 15min at 4°C, followed by filtration of the liquid supernatant through 0.2μm syringe filters (Pall Corporation, New York, United States). The filtrate (10μl) was subsequently injected into the third *S. frugiperda* larvae using a Hamilton Microliter syringe (705N) to introduce the AINV into the host. Control hosts were injected with an equal volume of sterile water. The *S. frugiperda* larvae were reared in room, followed by detection of AINV infections in the moths using AINV specific primers, AINV_72F and AINV_44R (Table 1). The *S. frugiperda* larvae were reared in room, followed by detection of AINV infections in the moths using AINV specific primers, AINV_72F and AINV_44R (Table 1). AINV titers in *S. frugiperda* were quantified based on the ORF2 (polyprotein) copies and then normalized with *S. frugiperda* β-actin gene copies. The qPCR reactions were performed in a CFX384 Touch^™^ Real-Time PCR Detection System (Bio-Rad, United States) using the SYBR Green method. The cDNA used in qPCR reactions was synthesized using the PrimeScript^™^ RT reagent Kit with gDNA Eraser (Takara). The reactions were carried out in a 10μl volume containing 5 ul of 2×TB Green^®^ Premix Ex Taq^™^ (Takara), 0.4μl of each primer (10μm), 1μl of cDNA, and 3.2μl sterile water. The qPCR primers used are listed in Table 1. AINVQF and AINVQR2 amplified a 102bp fragment of the AINV, while SFACTINF and SFACTINR amplified a 156bp fragment of the *S. frugiperda* β-actin gene. The qPCR reactions for quantifying AINV titers in infected male and female *S. frugiperda* larvae were done in triplicate. Four replicates of AINV infected larvae were used in these qPCRs.

### Effect of AINV Infection on *S. frugiperda*

Six fitness indices, including larval period, pupal period, pupal weight, pupation rate, eclosion rate, and sex ratio, were measured in AINV positive and negative *S. frugiperda* populations to uncover the effects of AINV infection on the novel host. The GraphPad Prism 8.3.0 software was then employed to analyze and visualize the data for the six indices.

RNA-seq was also employed to reveal the effects of AINV infection on the gene expression of female *S. frugiperda* adults. The first day hatched moths were subjected to RNA-seq, and three replicates of AINV positive and negative females were used following the aforementioned sequencing procedure. The HISAT2 (2.0.4; Kim et al., 2015) alignment program was used to map the obtained reads to the *S. frugiperda* published genome (ZJU_Sfru_1.0, GCF_011064685.1). Then, the mapped reads were assembled using the StringTie (Version: 1.3.4) sequence assembler (Pertea et al., 2015), followed by merging of the transcripts using gffcompare (Version: 0.9.8).^3^ The ballgown R package^4^ was subsequently used to estimate the expression levels of all transcripts and to detect the differentially expressed mRNAs by calculating FPKM (Fragments Per Kilobase Million; FPKM = [total exon fragments/mapped reads(millions)×exon length(kB)]). The edgeR R package (Robinson et al., 2010) was employed to filter the differentially expressed genes (DEGs) at a threshold absolute log_2_ (FC, fold change)≥1 and false discovery rate (FDR)<0.05. The DEGs were shown with volcano plot, which was visualized using the ggplot2, ggrepel, gridExtra, and ggthemes R packages. Principal component analysis (PCA) of the samples was further determined with the gene expression matrix. The analysis was then visualized using the stats R package. GO and KEGG enrichment results of the DEGs were visualized using the clusterProfiler R package (Yu et al., 2012), while the heat maps were visualized using TBtools (Chen et al., 2020).

## Results

### RNA Virus Detection in *A. ipsilon*

There were 62,933 unigenes within the *A. ipsilon* transcriptome. Two virus-originated fragments were detected as: unigene-23,909 (5,239 nucleotides) and unigene-23,910 (6,052 nucleotides). Blastp searches revealed that both unigenes were similar to the protein sequences of a Nora virus isolated in *Helicoverpa armigera* (HaNV, accession no: MK033133). These findings suggested that the unigenes probably originated from a Nora virus member and was tentatively named AINV. Supplementary Figure S1 showed the alignment of the two unigenes. The overlapping region between unigene-23,909 and unigene-23,910 was subsequently amplified, and the sequences were manually verified using specific primers (Table 1). Furthermore, following the methods described by Yang et al., (2019), we enriched the virus particles in AINV positive population and then observed them with a transmission electron microscope. The result was provided in Supplementary Figure S3.

### AINV Genome Characterization, Phylogenetic Analysis, and Quantification in *A. ipsilon*

**Figure 1:**
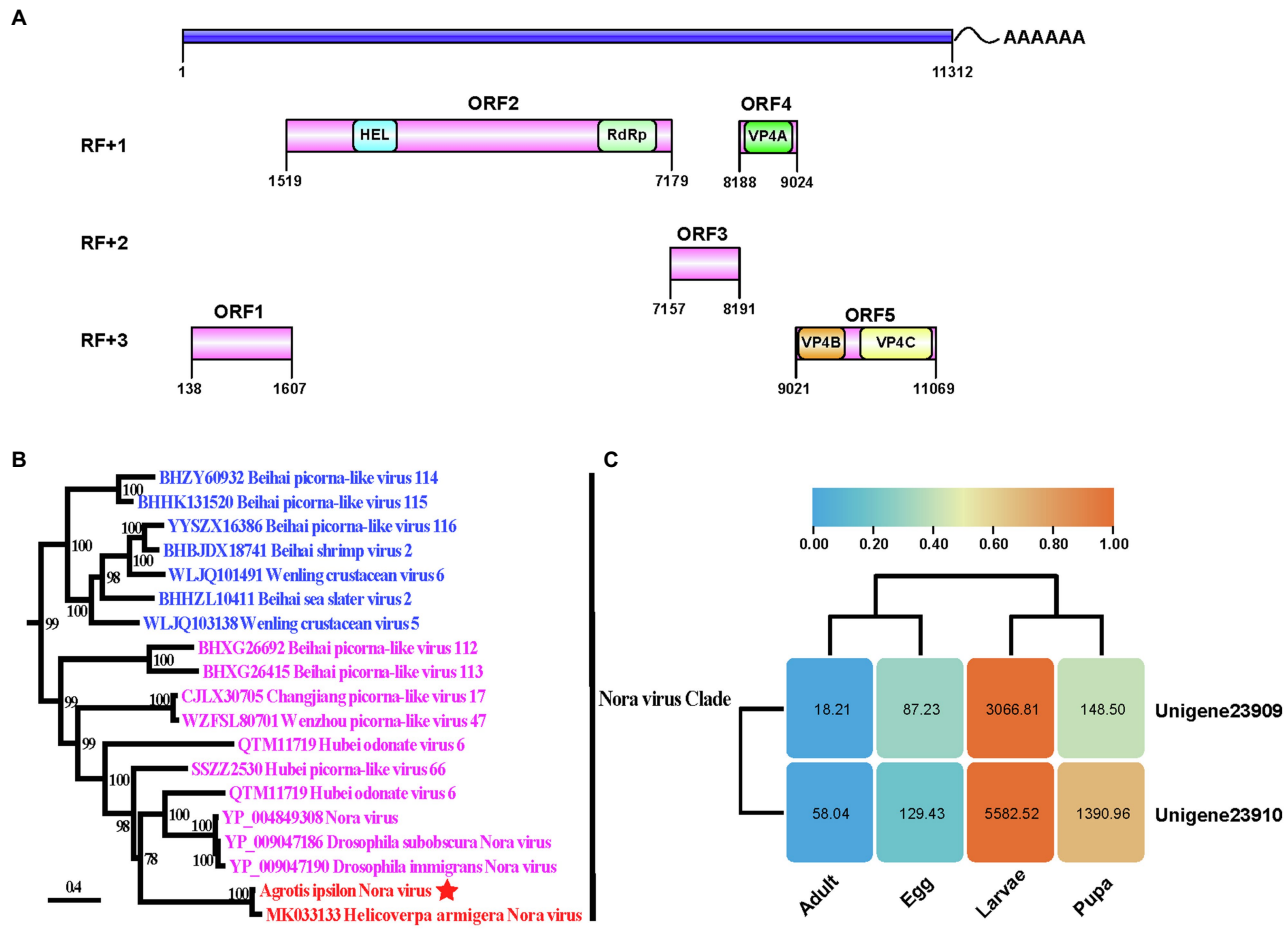
The genome characteristic and phylogenetic analysis of *Agrotis ipsilon* Nora virus (AINV). **(A)** The genome organization of *A. ipsilon* Nora virus. HEL, RNA helicase; RdRp, RNA-dependent RNA polymerase, and VP, structural protein. The genome structure is visualized by IBS program (Liu et al., 2015). **(B)** The maximum likelihood phylogeny of the Nora virus clade. *A. ipsilon* Nora virus is underlined. Numbers near nodes indicate ultrafast bootstrap values. The bar indicates the estimated number of substitutions per site. **(C)** The expression patterns of unigene-23,909 and unigenes-23,910 among *A. ipsilon* developmental stages. The numbers in the round rectangle indicate the FPKM (Fragments Per Kilobase Million) values. Log2 FPKM values are indicated in the clustered heat map.

The RACE alignments (data not shown) indicated that the complete genome of AINV was 11,312 nucleotides in length, excluding the poly(A) tails (Figure 1A). The locations of unigene-23,909 and unigene-23,910 on AINV genome were position 25 to position 5,263, and position 5,261 to position 11,312, respectively. The genome shared 85.69% nucleotide identity with HaNV with a 96% coverage. The ORF finder results suggested that AINV had a (+) ssRNA genome containing five ORFs. The conserved replication polyprotein domains were in ORF2. Comparison of the replication polyproteins of AINV and HaNV revealed a 14.60% genetic divergence between them. In the same line, sequences homologous to the three structural proteins of *Drosophila* Nora viruses, named viral protein (VP) 4A, VP4B, and VP4C (Ekstrom et al., 2011), were also detected in ORF4 and ORF5 of the AINV.

The LG substitution model with unequal amino acid frequencies (+F) and rate variation among sites (+R10) was selected in the phylogenetic analysis. The results strongly supported the monophyly of the Nora virus clade (UFBoot=99; Supplementary Figure S2). Furthermore, AINV shared a sister relationship with HaNV (UFBoot=100) within the Nora virus clade (Figure 1B) and further clustered with Nora viruses detected in *Drosophila* and Odonata (UFBoot=78). These findings confirmed that AINV was a new Nora virus member.

Both unigenes exhibited the same expression pattern in *A. ipsilon*, with higher expression levels in larvae and pupae than in eggs and adults (Figure 1C). These findings suggested that AINV titers varied among the developmental stages *of A. ipsilon*, with the larvae and adults having the highest and lowest AINV titers, respectively.

### AINV Novel Host Transmission

**Figure 2:**
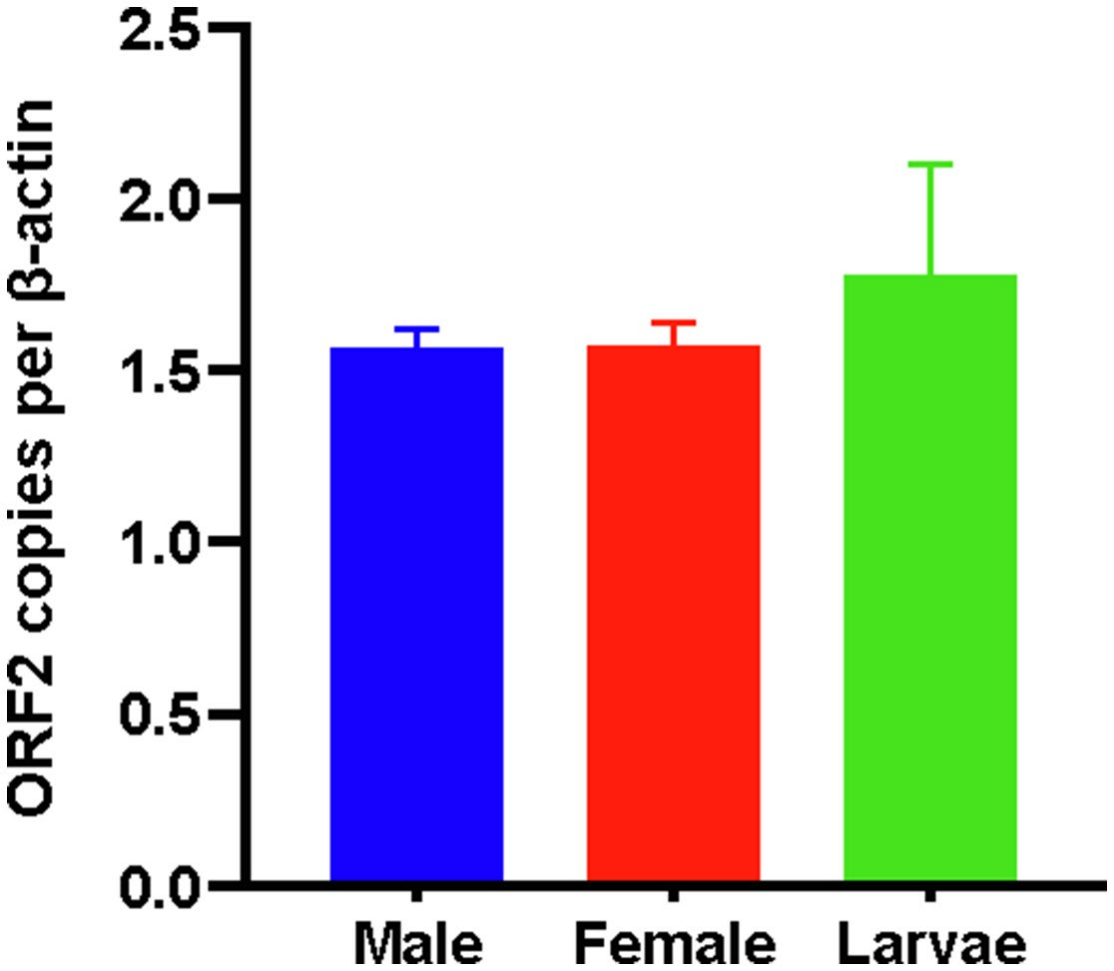
The AINV titers in *Spodoptera frugiperda* corresponding to the viral gene copies.

The AINV liquid was injected into 45 third *S. frugiperda* larvae, while another 45 larvae were injected with sterile water to act as the control. The adults of these larvae were then harvested and subjected to AINV detection using specific primers. Notably, all the adults in the AINV liquid injected group were positive for AINV, but none was positive in the controls. These findings suggested that AINV had a highly accurate horizontal transmission rate even between different hosts with the liquid injection method. Further quantification of the AINV based on the ORF2 polyprotein copies and subsequent normalization with *S. frugiperda* β-actin gene copies revealed the highest AINV titers in the larvae (1.77±0.32, *n*=4). In addition, male (1.56±0.05) and female (1.57±0.07) *S. frugiperda* had almost similar AINV titers. Similarly, there was no significant difference in AINV titer between the larvae and adults (*F*=1.086, *p*=0.388; Figure 2).

### Effects of AINV on Fitness and Gene Expression of *S. frugiperda*

**Figure 3:**
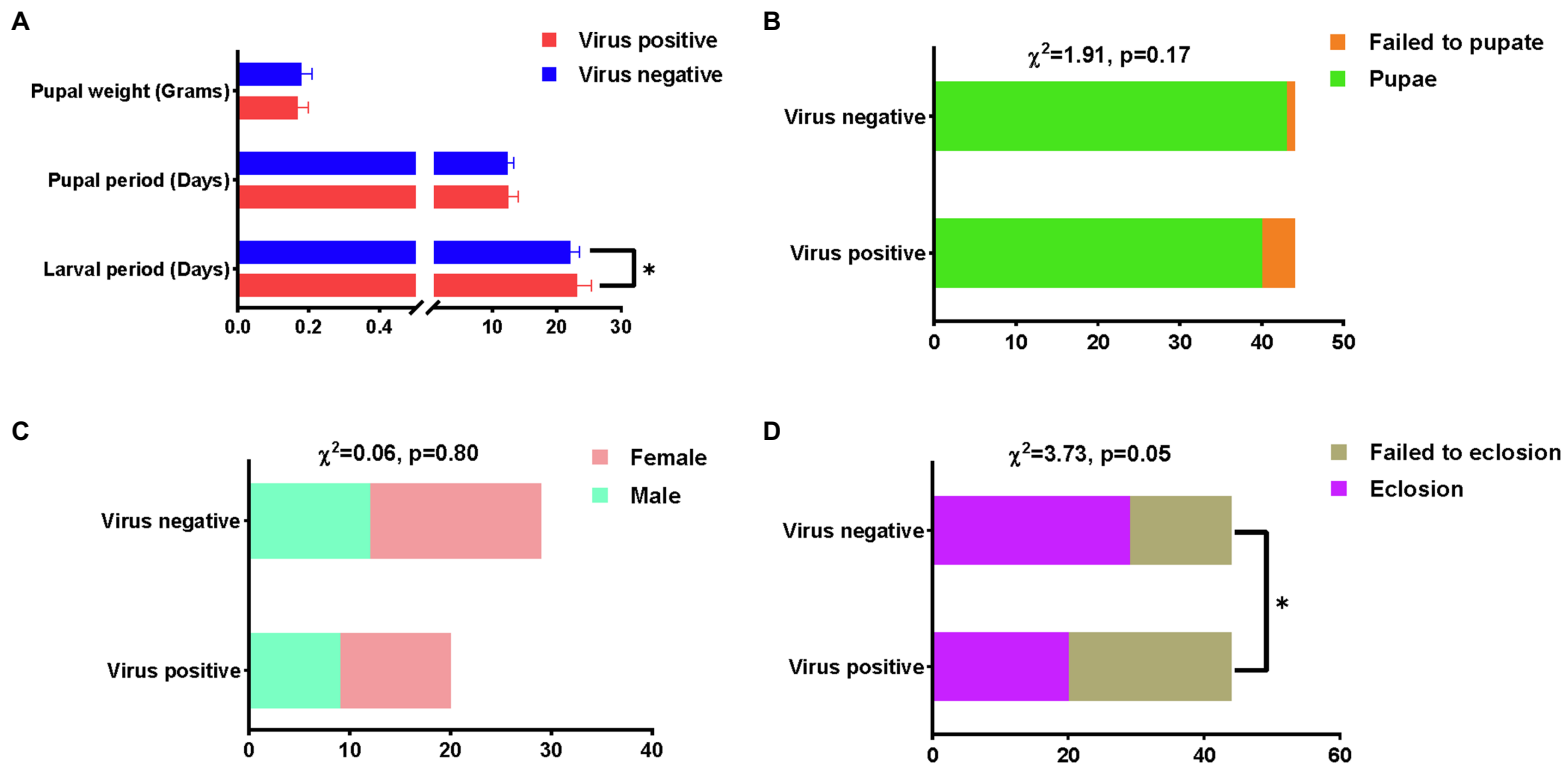
The comparative results of fitness measurements between AINV positive and AINV negative *Spodoptera frugiperda* populations. **(A)** The measurements of the larval period, pupal period, and pupal weight. **(B)** The measurements of pupal rate. **(C)** The measurements of sex ratio. **(D)** The measurements of eclosion rate. ^*^ Indicates value of *p* lower than 0.05.

AINV infection significantly increased the larval (*p*<0.05) and pupal growth periods and decreased the pupal weight (Figure 3A). There were no significant differences in the pupae rate and sex ratio between the AINV positive and negative populations (Figures 3B,C). However, the AINV negative population had a higher pupae rate than the positive population. Similarly, the moth eclosion rate was significantly decreased in the AINV positive population (*p*<0.05; Figure 3D). Moreover, we also observed that the AINV positive females laid about average 30 eggs that are much less than the AINV negative females, which can averagely lay more than 500 eggs under room conditions. It indicated that AINV infection would significantly affect the reproductive capacity of *S. frugiperda* females.

**Figure 4:**
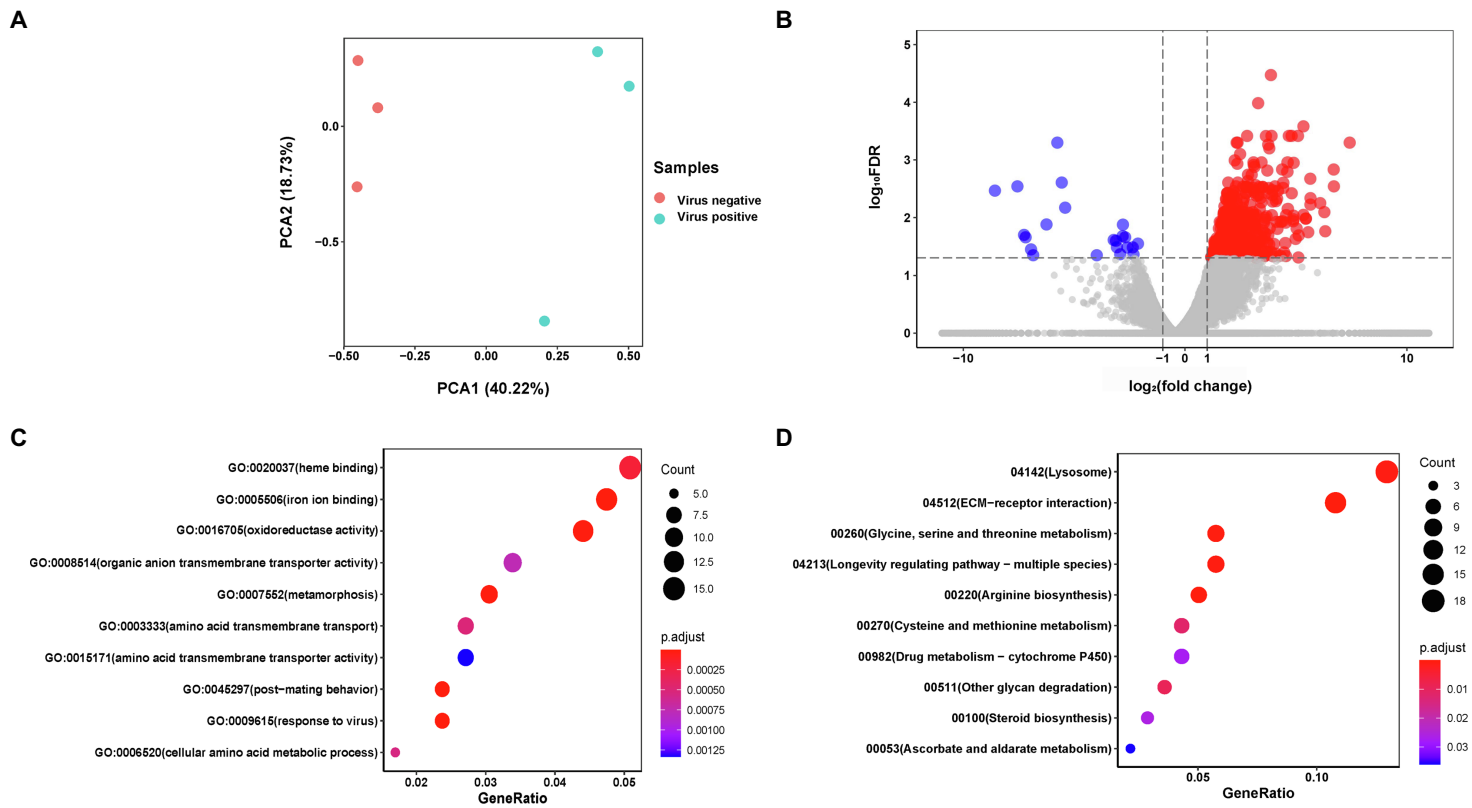
The summary of RNA-seq results in AINV positive and AINV negative *Spodoptera frugiperda* females. **(A)** Principal component analysis (PCA) of the samples using the gene expression matrix. **(B)** Volcano plot of differentially expressed genes (DEGs). **(C)** The GO enrichment of DGEs. **(D)** The KEGG enrichment of DEGs.

**Figure 5:**
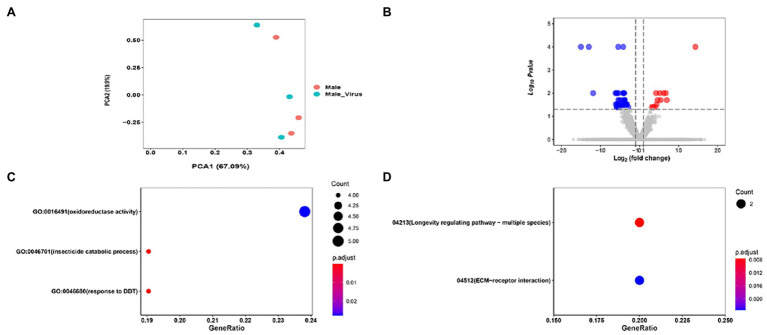
The summary of RNA-seq results in AINV positive and AINV negative *Spodoptera frugiperda* males. **(A)** PCA of the samples using the gene expression matrix. **(B)** Volcano plot of DEGs. **(C)** The GO enrichment of DGEs. **(D)** The KEGG enrichment of DEGs.

PCA results of AINV positive and negative adult subjected to RNA-seq revealed that the AINV positive and negative replicates were well distinguished in females (Figure 4A), but not in the males (Figure 5A). This finding suggested that AINV infection strongly changed the gene expression pattern in the *S. frugiperda* females, nor the males. Hence, in this study, we mainly uncovered the effects of AINV on the gene expression patterns in *S. frugiperda* females. However, the analyses of males were provided in Figure 5.

**Figure 6:**
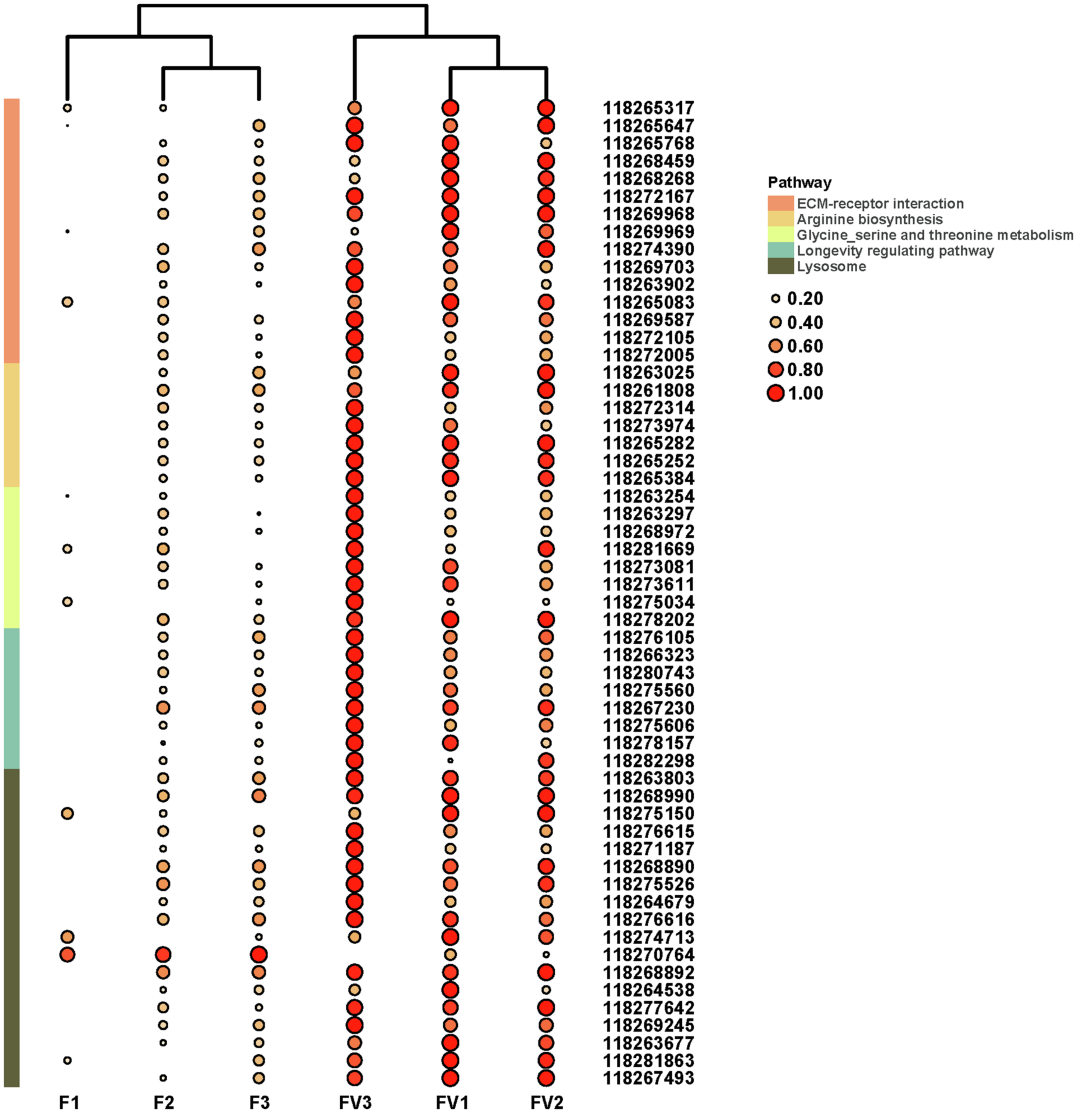
The expression patterns of DEGs related to the significantly enriched pathways. F1-3, AINV negative *S. frugiperda* females; FV1-3, AINV positive *S. frugiperda* females. Log_2_ FPKM values are shown by the color and area of the circles. Log_2_ FPKM values are further scaled by row with zero to one method, and then shown by the color and area of the circles.

Based on the log_2_FC>1 or log_2_FC<−1 and FDR<0.05 threshold, 627 unigenes were filtered as DEGs. Among them, 26 were significantly downregulated while 601 were upregulated in AINV positive females compared to the AINV negative females (Figure 4B). The enriched GO terms of the DEGs included a response to virus infection, iron-binding, amino acid metabolism and transport, and insect metamorphosis and mating process (FDR<0.05; Figure 4C). Similarly, the enriched KEGG pathways of the DEGs included the lysosome, ECM-receptor interaction, amino acids biosynthesis and metabolism, and longevity regulating pathways (FDR<0.05; Figure 4D). Moreover, all the DEGs grouped into these KEGG pathways were upregulated in AINV positive females except DEG118270764 (Figure 6).

## Discussion

The Nora virus is a single-stranded, positive-sense RNA virus that was first reported in two *Drosophila* species: *D.melanogaster* and *Drosophila simulans* (Habayeb et al., 2006). Thereafter, metagenomic analyses have revealed novel Nora-like viruses in *Drosophila* (van Mierlo et al., 2014; Medd et al., 2018), *Nasonia* (Oliveira et al., 2010), honey bee (Remnant et al., 2017), and diverse environmental samples (Shi et al., 2016). Recently, Nora-like virus infections were also detected in two lepidopteran pests: *S. exigua* and *H. armigera* (Yang et al., 2019). Herein, AINV clustered with the *H. armigera* Nora virus with robust supports and grouped into the Nora virus clade suggesting that *A. ipsilon* is the third lepidopteran natural host of Nora virus. Nora virus shares a conserved picornavirus-like helicase-protease-replicase (H-P-Rep) cassette and has a unique genome organization pattern (Habayeb et al., 2006; Ekstrom et al., 2011). The taxonomic position of the Nora virus is further confirmed by the recent structure analyses of its capsid proteins (Laurinmaki et al., 2020). The virus has a relatively large RNA genome, comprising more than 11,000 nucleotides in length (Habayeb et al., 2006; Laurinmaki et al., 2020). AINV has 11,312 nucleotides and which include five ORFs. The ORF2 of AINV encodes the picornavirus-like replicative cassette, while ORF4 and ORF5 encode the three structural proteins. The AINV ORFs have short overlaps, indicating that ribosomal frameshifting maybe involved in the expression of its proteins (Dreher and Miller, 2006).

Nora virus can cause persistent infections in *Drosophila*, and these infections are not affected by the *Drosophila* RNAi pathways (Habayeb et al., 2009b). The *Drosophila* Nora virus is horizontally transmitted through the fecal-oral route, causing mild effects on the fitness of infected flies (Habayeb et al., 2009a). A recent study postulated that geotaxis dysfunction is a phenotypic hallmark of *Drosophila* Nora virus infection, which causes the infected flies to significantly lose their climbing ability (Rogers et al., 2020). Notably, the Nora virus is horizontally and vertically transmitted within *H. armigera* populations, with unclear pathogenic effects on the infected moths (Yang et al., 2019). Similarly, AINV stably infects the *A. ipsilon* population, with no clear negative effects on the fitness of *A. ipsilon* (data not shown). As such, AINV is probably a mutualistic virus in *A. ipsilon*. Novel partiti-like viruses can horizontally transfer among congener lepidopteran hosts and subsequently cause deleterious effects on the new hosts (Xu et al., 2020). These findings strongly suggest that the novel viruses are potential biological resources for pest management. *S. frugiperda* is a destructive crop pest discovered in China in 2018 (Sun et al., 2021). Herein, AINV was injected into *S. frugiperda* and established stable infections, indicating that AINV can horizontally transfer among the Noctuidae hosts. Our previous study indicates that relative to the oral transmitted pathway, microinjection has its own advantage in the horizontal transmission of novel insect viruses (Xu et al., 2020). Hence, in this study, we establish the AINV infection *S. frugiperda* population by microinjection. However, we also notice that Nora virus can horizontally transmitted in its original host with perfect transmitted rate, by sharing the virus contamination food (Yang et al., 2019). It implies that AINV is probably can also be transmitted among *A. ipsilon* individuals through food-borne transmission route. AINV titers in *A. ipsilon* and *S. frugiperda* varied among its host developmental stages, with the larvae having the highest titer. AINV infection also had some side effects on the fitness of its new host, *S. frugiperda*. It significantly extended the *S. frugiperda* larval period but significantly reduced its moth eclosion rate. Moreover, after AINV infection, *S. frugiperda* pupal weight loss, it indicates that AINV and *S. frugiperda* lack a long co-evolutionary history which causes AINV to be a parasitic virus of *S. frugiperda*.

Previous studies postulate that the *Drosophila* Nora virus structural protein has RNAi suppressive activity in its natural host (van Mierlo et al., 2012, 2014). Herein, RNA-seq analysis revealed a higher expression of Argonaute-2 gene (AGO2) in AINV positive *S. frugiperda* females than the negative ones (Fold change=1.47, FDR>0.05), indicating that AINV stimulates the RNAi antiviral defense in the new host. Nora virus infection upregulates immune genes in *Drosophila* (Cordes et al., 2013; Lopez et al., 2018). GO and KEGG enrichments herein revealed no immune-related pathway in response to AINV infection. However, some potential antiviral pathways, such as ko04142 (lysosome), were uncovered (Du and Jin, 2017). Notably, ko04512 (glycine, serine, and threonine metabolism; Ding et al., 2019) and ko00260 (ECM-receptor interaction; Guo et al., 2015) have been reported to respond to animal virus infections. Herein, the DEGs are mainly enriched in the amino acid-related pathways, indicating that AINV infection affects the amino acid metabolism in *S. frugiperda*.

## Conclusion

This study reports a new Nora virus infecting *A. ipsilon* and provisionally names it AINV. Its genome has 11,312 nucleotides, which include five ORFs. AINV was successfully transmitted into a novel host, *S. frugiperda,* through injection, causing stable infection, suggesting horizontal AINV transmission among moths of the same taxonomic family. Furthermore, the fitness measurements and RNA-seq analysis showed that the AINV infection was deleterious to *S. frugiperda* and mainly mediated by antiviral and amino acid metabolism-related pathways.

## Data Availability Statement

The datasets presented in this study can be found in online repositories. The names of the repository/repositories and accession number(s) can be found at https://www.ncbi.nlm.nih.gov/, PRJNA742481 and PRJNA76097.

## Author Contributions

LT: formal analysis, investigation, data curation, and writing—original draft. GR: formal analysis and writing—original draft. WY: resources. CS: investigation. YG: supervision. MX: project administration. LH: conceptualization, formal analysis, data curation, and writing—review and editing. All authors read and approved the final manuscript.

## Funding

This work was supported by the Fund for Distinguished Young Scholars from the Henan Academy of Agricultural Sciences (grant no. 2020JQ05) and the National Natural Science Foundation of China (31772520; 31702057; and 31601897).

## Conflict of Interest

The authors declare that the research was conducted in the absence of any commercial or financial relationships that could be construed as a potential conflict of interest.

## Publisher’s Note

All claims expressed in this article are solely those of the authors and do not necessarily represent those of their affiliated organizations, or those of the publisher, the editors and the reviewers. Any product that may be evaluated in this article, or claim that may be made by its manufacturer, is not guaranteed or endorsed by the publisher.

## Footnotes

^1^ https://www.ncbi.nlm.nih.gov/orffinder/

^2^ https://www.ncbi.nlm.nih.gov/Structure/cdd/wrpsb.cgi

^3^ http://ccb.jhu.edu/software/stringtie/gffcompare.shtml

^4^ http://www.bioconductor.org/packages/release/bioc/html/ballgown.html

## Supplementary Material

Supplementary Figure S1 The sequence alignment of unigene-23909 and unigene-23910.

Supplementary Figure S2 Maximum likelihood phylogeny of *Picornavirales* and *Caliciviridae* with RdRp protein sequences. The Nora virus clade was highlighted. Numbers near nodes indicate the ultrafast bootstrap values. The bar indicates the estimated number of substitutions per site.

Supplementary Figure S3 Electron microscopy of purified AINV particles.
