# Supplementary material for: The Novel Agrotis ipsilon Nora Virus Confers Deleterious Effects to the Fitness of Spodoptera frugiperda (Lepidoptera: Noctuidae)
Source: Front Microbiol. 2021 Nov 15;12:727202. doi: 10.3389/fmicb.2021.727202 (PMC8634655; doi:10.3389/fmicb.2021.727202)
Supplement: Supplementary Figure S1 — The sequence alignment of unigene-23909 and unigene-23910. [file Data_Sheet_1.PDF]

```
Unigene23909 ATAATATGTGTGGTTATCTAAAAATAAGAAATAAGTCTTGGCCCCTAGTGGTTATCTAAAAACAAGAATAAAATCTTAGC
Unigene23910 --------------------------------------------------------------------------------

Unigene23909 CGTTCACGTTGTAATTTTTGTGCGCATTGTTTTATGTTTATGTCTAAACAACATAGTAATCAGGTGGCTGACGGTTCAGG
Unigene23910 --------------------------------------------------------------------------------

Unigene23909 CTTAGTGACCGTAGTTTCACCTCAACATGCTAACAAGCCAAACGACTTTAGCGAGAATAAGGACTTTTCAAAATCTAACA
Unigene23910 --------------------------------------------------------------------------------

Unigene23909 TGGTTATTACACTATATGATTTAGGTCTTCGCTCACGCGATGACGTTAAACAATTAATTGAGACTGGATTATTCCCGGAT
Unigene23910 --------------------------------------------------------------------------------

Unigene23909 CAATTACTAGCACTTAAGAATGAGTTGACAGTAGCTCAGCTTAATTTACCGAAGTTTAGTCGACGATATCAGACAAATGA
Unigene23910 --------------------------------------------------------------------------------

Unigene23909 GCTTTCCCGTAAAGCACAATTGTCTAATATTATTGACGGTGAATTAAACGGATTTACTGTTACTCAAGGTGTTGCGCATA
Unigene23910 --------------------------------------------------------------------------------

Unigene23909 CGACGAGCTTTAAAGCTCAGTCCAATACGCAACTTAAAAAACGAAGAGCGAAGTTAACGAAAGCAAACTTTGCTTTTGGT
Unigene23910 --------------------------------------------------------------------------------

Unigene23909 TTAGATGGTTTACCCATAACCCAGCACGGTAATATGACAGCTATTAATGAAGATACTAATCTTGTAGATGAGAGCTTATG
Unigene23910 --------------------------------------------------------------------------------

Unigene23909 TTCTGATACTACATGTCAATCTGAAACATCTGTCCAACTGGAAGTGGAGGAACCACAAATGATAGAAACTTCATCTGTTA
Unigene23910 --------------------------------------------------------------------------------

Unigene23909 TTTGTGAATCTTCTACTCCCAGTGAGTACAATGACGTTGATGCCATTTGTGATTTGATTGATAAATTGGAGAAATCCGAT
Unigene23910 --------------------------------------------------------------------------------

Unigene23909 TTAGCGATCGTTTACCGATATGCTCAACGAAAATATGACCAAACTCCCGGACTTCCTGTGGTTCCTACTAAGAAACCGCG
Unigene23910 --------------------------------------------------------------------------------

Unigene23909 TAAAAAGAAGAAGAAGCAGACGGTAGAAGCACCACCTGTTCTTTCGGAAAAGGAGTATCCTTCTTTACAGAAGGAAATTC
Unigene23910 --------------------------------------------------------------------------------

Unigene23909 CTCTTCCGGAAGAGACGGTATCCGTGGAAACACTTGACCTTCCTAAGAAAAAGAAGAAGCGGAAGCAGAGAAAAGCACAA
Unigene23910 --------------------------------------------------------------------------------

Unigene23909 ACTCCCTTGTTTGGAGGAGCTGCTGATTCTGGTGTAGAAATTGAAGAACACGGAAATGTTGTTTCAAAAGAAGCAACCTC
Unigene23910 --------------------------------------------------------------------------------

Unigene23909 TTCGGATGAAGTTTCAATACCAGCATCACCAGTTAAGAAGAAGAAGATTGTCTTTAAACCTTTTATAGACCAATCATCTT
Unigene23910 --------------------------------------------------------------------------------

Unigene23909 CTGACCAACCGAAATTTGATTTTGTGTTACCGGCAAAAAGTAAACAGGGTAGTAGTATACATCAGTGTCCTATTGCCAAA
Unigene23910 --------------------------------------------------------------------------------

Unigene23909 TACCCCCTACATCTCTGGAAGCGAGCTTTGAACAAGTGGCCAAACCACTCAATCGAAGAAGTTGCCGAAGCAGAGTTTAA
Unigene23910 --------------------------------------------------------------------------------

Unigene23909 GCGGATGGTTAAATCCTCATTGATACACTGGACCCTCCAGTGGAAGCGTAGTAGCACCGATCGCAACCCATGGTTAGCCG
Unigene23910 --------------------------------------------------------------------------------

Unigene23909 ATTGGAACGCTACTATTCGTCAGCTTATGAAGTATGACATGAAAGAATGGTACAATGCATACCAAGCTTTCAGAGATCGT
Unigene23910 --------------------------------------------------------------------------------

Unigene23909 GCAACTAGCTATGCTGAAAAGAACTCAAACCCACAGTGGGCAACTGAACCTAAACAAAACTAATATGACAGTAACACAAA
Unigene23910 --------------------------------------------------------------------------------

Unigene23909 GAATAATAAATCAATGTGTGCTCATAAACCAAACGGTCTTATGGGCACTCCAAAGGAAAGAGTATGCGCTCCTCGGAGTT
Unigene23910 --------------------------------------------------------------------------------

Unigene23909 ATGCTTATTCTAGTAGGGTTAATCTTTCACGTTCCGTTAACGCATACATTCATGGAAATGTTTATGTATACGATGATTGT
Unigene23910 --------------------------------------------------------------------------------

Unigene23909 TCGTTTTTTGATGGGTAGTCCCGGTGAAGTTATTTCAAAATTTTTCGAAGAAATAACAAATTATTCACAGACTTTCGTTA
Unigene23910 --------------------------------------------------------------------------------

Unigene23909 CGCAAGTGAAGGAAGTTACTGTAGATGCTGTTAGTGAAGGTTTACGTGATATCCGAGATGCTGCAACACCAGATGTTCTT
```

```
Unigene23910 ------------------------------------------------------------

Unigene23909 AGCTTTAAGAATATCTCCACCAATTTCTTGGATCATAAGATCGGTATTGCTACTTGCGTGGCATCAGCCTCGCAGGCTAG
Unigene23910 ------------------------------------------------------------

Unigene23909 TAACGCCGTTCAAGTTGGTTCAGAAATTATTAAGGTAGGAAGTATGCTCGGTCTTGAAAAATCGCTACTTGGTAATGTTG
Unigene23910 ------------------------------------------------------------

Unigene23909 TAAACGCTGTGGTACACCGCGGTGCTGACATGATCTTACCTCGTATTGATGAACACGGTCTAGAAGATTGTGTTCCGCTT
Unigene23910 ------------------------------------------------------------

Unigene23909 GTCGCTACAGCCGCAGCGATGGCTGGAACAGAATTTTTGGACTTTAATATCGAAACCACGCTTAAACGCGCAGCACAGAA
Unigene23910 ------------------------------------------------------------

Unigene23909 CACCAAAGCCATGGAAACCCTTGTTGCACACACTCGCAAAGTGTGCGAACAGAGTGGTATCATACAACGCCCCGGTTATA
Unigene23910 ------------------------------------------------------------

Unigene23909 CAGAATTGATGAGGATTAATGAACGCATTGAAAAACTGCGTGATGAAACTCTGTGGATGTCCCAAATGCTAGGTATAAAT
Unigene23910 ------------------------------------------------------------

Unigene23909 GGTTCCGTGTTTAACACCCCTGATGGAAAGAAGAGGGTTATTGAATACAAACATGAAATCGATGCTCTTATGCGTGAGCT
Unigene23910 ------------------------------------------------------------

Unigene23909 AAAGAGTGTCGGTCAAAGTACGGTTAAAGATACATCTTTATACACAGAACTTCATTTTTACTTGGTTAAATCAGTGGATA
Unigene23910 ------------------------------------------------------------

Unigene23909 TGTTGTCACAAATTAGTGCCATACAGGCCTCTAGTGGTCTTCGTATGGTACCGGTAGGTATCAACATATTTGGTGATTCC
Unigene23910 ------------------------------------------------------------

Unigene23909 CAAATCGGTAAGTCTATGTTGGTTCAGGAAATCCACAAAAGAGTGCAACAGCAGTGTTTAGCTGAACATGTCAATCTACC
Unigene23910 ------------------------------------------------------------

Unigene23909 TCGTGTGGGTCATTGGCAAATTTGGCATATGCAACAGCGAGATGAGTTTGATACTAATTATTACAGTCAAGAGGTAGCAT
Unigene23910 ------------------------------------------------------------

Unigene23909 ACTGCGATGATGGCTGGAGTGATAAAACTAATAAAGATCATCAACAATGGCTTAATTTTATTAGTCCAGCCCCTATCGGA
Unigene23910 ------------------------------------------------------------

Unigene23909 ACTGTGCAAGCGCAGTTGAATCAGAAAGGTGCACCTTTTCAGGCCAACATGTGTATAGTTACATCAAACACACTCCCTCG
Unigene23910 ------------------------------------------------------------

Unigene23909 TACTAGCATTGCTATAAACAATGTCAATGCGCTCCATGCGCGTTTCCCTGTTTGTATCAAGGCTACACGGGTGAGCAACC
Unigene23910 ------------------------------------------------------------

Unigene23909 CCTCACAGGTTTATGACCCCGATTTTAAACATCTCAAGTTTAGTGTCGGAACAATGAGTATGCAACTCAACCCGGAAACT
Unigene23910 ------------------------------------------------------------

Unigene23909 TCAAATTGCGTTGAGCTTGACCTGAATGCTATCACAAAAATGATAGTTCGTCACTTGCGTAAGAATCTTGAACTGTATCA
Unigene23910 ------------------------------------------------------------

Unigene23909 AAGTCAGACTAATATTATTAGTCAGGCCCTCGATGTAGTCGAACAGAATTCTGGCGCACCTATTGAACAACATGCAGATC
Unigene23910 ------------------------------------------------------------

Unigene23909 GTCACGAACTACAGCCAGTTCGATCGCAAGGCATGCCTCAACTAAACGGAATGACGCTTAAGCAGATCTTGGGTTTAAAA
Unigene23910 ------------------------------------------------------------

Unigene23909 CGCGAGTGGCGAAGTGCTCGTAATCAGCGTCCAATTATCGGATTGAATCAATTTGGTCGTTGGACTCAGCTCTTGCGCCA
Unigene23910 ------------------------------------------------------------

Unigene23909 TCGTACAACAAATCAGACTGTGACCGAATGGGTCGCTGGATCTCCGGATCTAACTGCCTTTGAATTTATTTCTGCTCTAG
Unigene23910 ------------------------------------------------------------

Unigene23909 GCATATGGATAGTTCCTCCCGAACATATTGAAGAATTCAAGATTGCATTTGCTGAACAACCAATTATACGTGTTGAAGAT
Unigene23910 ------------------------------------------------------------

Unigene23909 CATTATGAGACTCCTTATTATTGGGGTCCACGTATAGATGGTGGTTCAATGCTTGTTGTTCATGTCCCGGCTGTTGATTT
Unigene23910 ------------------------------------------------------------

Unigene23909 GTTAATTGATAACTTTTTAACATCCACGGGTATGGACCATGAGATTCAAGAGTTTCATCAAGGAATTGCTTCGATGGCTA
Unigene23910 ------------------------------------------------------------
```

```
Unigene23909 TTACTATTGGAGTACAAATCTTTCTAGGTTGTGTTGAACCTAGTGTCGCCCTCGAACTGTTAGGTACAGCGGTTTCAGTT
Unigene23910 --------------------------------------------------------------------------------

Unigene23909 GCCACTTGGTCCCCTATGTTGCGTGAAGTACCTTGGTACAATGCATTTAGGCATAGCGCGGGTCTGGTTGCCGCCAAAAC
Unigene23910 --------------------------------------------------------------------------------

Unigene23909 CCTTGCTGTTGTGTTTTCACCATTTACTGCGCTTTATCAAAAGATTGACTCTCGTATTCGTGTAATTACGATGAAGATAG
Unigene23910 --------------------------------------------------------------------------------

Unigene23909 GAGATATTCTTGTTTCTATGAGTATTAGCTTGCTAGAACTTATAGGAGTCGAGATAACACCCACCACTGAGACTTGGTTG
Unigene23910 --------------------------------------------------------------------------------

Unigene23909 GTGGGTGCCTCCACTGTGGCTATTGAAATGTTGACAGCCGCTATCGTTGTTGTGATGGGTTACCTTCTTATACAATTGTA
Unigene23910 --------------------------------------------------------------------------------

Unigene23909 TAAAAAGATGACCACACCACCCGTCGACGATATCATCGAACATACACGATCTTACGAGGGTAAGGCTAAGAAAGATAAAT
Unigene23910 --------------------------------------------------------------------------------

Unigene23909 TAAAAAAGACAAAATCTGGTAAGATGATGAAAATTCGACAACATACCGAAAATGAGAGCATACGACCTTATTCACAAGAG
Unigene23910 --------------------------------------------------------------------------------

Unigene23909 AATGTTCAAGCCGATGTAGATTGGAAAGAGCATTTGTTGGATATGGTAGACGAAGGTTTGTCTAATGGTACTGACGCCTT
Unigene23910 --------------------------------------------------------------------------------

Unigene23909 ATATTACTCGTTTACTCCCAAAGATAGAACAGTAATATATGAGCATTTTAAGAATGATACCAACATGACCGGTACCGATT
Unigene23910 --------------------------------------------------------------------------------

Unigene23909 TTCTCAAATTTGAACGTATGCGTGTGCCAGTGGACGATCCCGATTCAGAGTTCGTTCAATTGGTTCAAGCTGTATGTTAT
Unigene23910 --------------------------------------------------------------------------------

Unigene23909 GAGTATGAGAAAACCGGTTCCCAGACCGAAGTTGAAAATTATCTGATAAATATTTTGGACAAAATATCCGGTCTCGTTGG
Unigene23910 --------------------------------------------------------------------------------

Unigene23909 TCAAACCAACATTAAAATTATTGTTTCAACTGAAATCATTGATGATGACACATATAGGACGTCATGTTTTATCTGTATTC
Unigene23910 --------------------------------------------------------------------------------

Unigene23909 CCAAAAGAGAAGAGAAAGGAAACAAATAATACATACACGTAAACATCTCAAAGAAGCGGAGGCATTTGTGGCAGTCTTA
Unigene23910 --------------------------------------------------------------------------------

Unigene23909 CAAGGCTGTGAACCTACTCAATCTGTGGATTCAATTATCCAACATGGTTTGCATGTAGACATAAAATCAGTTCGCAGTAG
Unigene23910 --------------------------------------------------------------------------------

Unigene23909 GTATGAGGTTTCTGTTACTGGATTTGTGTCATCTACGGCCGCGTGCCATGGATTTGGACTTGCTAGTGGAACCACCGTTA
Unigene23910 --------------------------------------------------------------------------------

Unigene23909 TTTGTAACTCTCATATTACTCGTAAAGGTGGTATTGTGTTGATTCGGCGACGCGGAGAAACAGACGGTCGATACCAGGTC
Unigene23910 --------------------------------------------------------------------------------

Unigene23909 GCGGAAGTTACGAATTTCGATAATCTCCGTGATCTCGCTTTTTGCCGTATACTTCCTGTCGATCAGGCTGCAAAGCCTGT
Unigene23910 --------------------------------------------------------------------------------

Unigene23909 GTCAATGCTTAGACCAGAGGCGCTCATGCCCAATCACCGTAACCTTACTTACCAACCGTTCCCGGATATGACCCAACATC
Unigene23910 --------------------------------------------------------------------------------

Unigene23909 TAATGTCGTTTAAAGATCTAATGGACTTGTATGGTTCAG-----------------------------------------
Unigene23910 ------------------------------------CAGCAGTTATGGTTGTGGCACCTCGCTCAGACTTAGTCATCACT

Unigene23909 --------------------------------------------------------------------------------
Unigene23910 GGTACTTTAGGGTATAGTGAAGGATGGGTCTCAACGGGTGCTCAAAGTTTTGAACGTAAACATCTCACGATTACAGGACT

Unigene23909 --------------------------------------------------------------------------------
Unigene23910 TGCTGTTACCCTCGAATTGCCTCAAAAGGGCGATTCGGGTGGTGTCATTATGGCTACTACTCGGAAAATGAACAACGGAA

Unigene23909 --------------------------------------------------------------------------------
Unigene23910 TTATTGGTATTTATACTGGAACTTCTAAGAGAACACTCCTAGGAGCTTATTTAGTTCGCGAGGATTTTGTAGAACTTCAT

Unigene23909 --------------------------------------------------------------------------------
Unigene23910 GGTTTAAATCAGGAAGATCCGTGGATGCAATTGATTGCACCTATGGCTACGCATAACGTCCCTGAAGGGCCTGCTGTTGT

Unigene23909 --------------------------------------------------------------------------------
Unigene23910 TGCTATTGGTGAATATCGATATGATAACGTACCTATTTCACGACCTCAGCATGTTAAGTGGGTTCGTGCTCCATGGGACT
```

```
Unigene23909 --------------------------------------------------------------------------------
Unigene23910 TCAAAGTCACGACAGCACCTCCACCACTTTCACCATATGATGAACGCATAGAAGTAGATTTACCAAAGAATGCTTTAGGC

Unigene23909 --------------------------------------------------------------------------------
Unigene23910 GAACCATCGCTCTTGATGAAACAAGTGCTACCATTGGCCCAGGCTATTCCAGATGTCAATCAACAACATTTGAATCATTA

Unigene23909 --------------------------------------------------------------------------------
Unigene23910 TGTCAAGTGCAGAGCTGAACAGCTTTCAGTGTTAATGCAGATTGCTAAGACACCGGAAGATATTGATTCCGTATTTGAAA

Unigene23909 --------------------------------------------------------------------------------
Unigene23910 ATGGTATAAATGGTCATCCAACAAATGTACATGTTAAAGGTATCTACACTAATTCTAGTAGTGGATTACCTTGGACTTCT

Unigene23909 --------------------------------------------------------------------------------
Unigene23910 AGCTCTGATCGTCAACTAAAAAAGGACATGCTGGATACTGACCCTCAAACCGGATATGTAACAGTAAATGAGAAAGGACA

Unigene23909 --------------------------------------------------------------------------------
Unigene23910 AGCGCTCCGAAAGCGGGTTCTCACTATTTTAGAGAGAGCAAATAATGGTGTGCGTAGTGTAGTGTTGGTTGCTTCGAAAC

Unigene23909 --------------------------------------------------------------------------------
Unigene23910 TGAAGGATCAATGCATTAAGATCAAGCACGTGAAGAATGGTAAAGTACGAGTGTTTCATGCAGTACCCGTTGAAAAGATC

Unigene23909 --------------------------------------------------------------------------------
Unigene23910 ATAGCAGATTCAGGTCTGTTTGGTAATTTCAAAGAAACCTACATGAATTTAGGACTAAAGCTCGATCATGCTATAGGTAT

Unigene23909 --------------------------------------------------------------------------------
Unigene23910 TGACCCAAACTCCCCTCAGTGGGGTGAGATAGTTAAACACCTCAAGCATGATCGATATTTAGATATGGATTTTACGCAAT

Unigene23909 --------------------------------------------------------------------------------
Unigene23910 ATGACAAACGTATGCATAGTCAATGTATGTATGCTTTTTATGACATAATACGAGCGGTGATCAAGGATAAGTGGTCCACA

Unigene23909 --------------------------------------------------------------------------------
Unigene23910 GCCCGTGATGTACTCGGCGACATGTCGATTAGATCCTGGGTTGTTGACTATAAGTCTGTTTATGAAACCTCACGAGGTAA

Unigene23909 --------------------------------------------------------------------------------
Unigene23910 TAAGAGTGGTGAATTTTTGACTACTGTTATTAACTCTGAGGTTAACCGCATGTACTCATGGTTAGCATGGGTGGCCATTA

Unigene23909 --------------------------------------------------------------------------------
Unigene23910 CTGGTCGCTCTGATTATGCAGAATGGCAAGAGAATGTCTCTCTTGTTACTTTCGGTGATGACAAGTTAGAAGGTGTGTCA

Unigene23909 --------------------------------------------------------------------------------
Unigene23910 GATGCTTTTGCTGACGTGTACCACTATAGAGCTCTAAAAGAATTTTATAGTGGTATAGGTCATACAATTACGTCTGACAC

Unigene23909 --------------------------------------------------------------------------------
Unigene23910 ACTTGATGAGTCTGGAGAACGGTTCTCTAAACTTGAAGAGCTTAAGTTTCTGAAACGGAGGTTCGTACCACTCGATGGTG

Unigene23909 --------------------------------------------------------------------------------
Unigene23910 TTATTACAGCCCCACTTGAGGTAGAAAGTATAGAGAATCCCTTTGTTTGGACTCAAATGATGGATTTTGATTTTGGTTTG

Unigene23909 --------------------------------------------------------------------------------
Unigene23910 TGGAAACCGCTTATCGCAGAATCGCTGTTAGAGGCGAGTCTGCATGGACGCGACTACTACGAATCGTTTGTCGAGATGCT

Unigene23909 --------------------------------------------------------------------------------
Unigene23910 CAAATGTTGTAAAAGCCGCGTATTGTTACGCGAGATTATTGATCTACTCCTGTCCGAGTACGATGATAGTAAGAAGAAAA

Unigene23909 --------------------------------------------------------------------------------
Unigene23910 TGGTAGAAAGATATGTCGGACACAGAATTTGGTAACTATGTGTTTCGTAATAACCTTACGGTATTCGAGGCATTATCAGA

Unigene23909 --------------------------------------------------------------------------------
Unigene23910 GTTAGATGTAGTTGACCTCCACACGGAAGTCGAAGCTAATAACGCATTAATAGTGCAAACACGTAGTGAACTTCAAGCTA

Unigene23909 --------------------------------------------------------------------------------
Unigene23910 CCGCTCGGTTACTTGATAATACTGTAGCGCATGTTAACACTATTGATGCTGTGTTGGTTGACGTCCGCGAAGAGGTTAAC

Unigene23909 --------------------------------------------------------------------------------
Unigene23910 TCACTAACATCTGAAATCAACTCTATTCAGACTGAGTTGCAAAATGTATCACAGCTGGCAACTTCCGCAAGTACTGCTGC

Unigene23909 --------------------------------------------------------------------------------
Unigene23910 AGATCATGCTGGTCTTTTAGCAGAGCAAGCAGCTACTCAAGCTGCAAATGCTTTAACTACTGCTAGGGCTGCTCAACAAG

Unigene23909 --------------------------------------------------------------------------------
```

```
Unigene23910 CGGCAGACTCAGCAGGTGCAACAGCGTCAACTGCTCTGTCAGAAGTGCAATCAGTGCGCAGTACAGCAAATGCTGCAGCT

Unigene23909 ------------------------------------------------------------
Unigene23910 ACTGCAGCGAGTGCTGCATCAACATCAGCTAATAACGCTCGATTAACTGCAGATTTGGCTCAGTCAACTGCTAATATGGC

Unigene23909 ------------------------------------------------------------
Unigene23910 TATGACGCGAGCTGACCAGGCTCGGACAACTGCTAACACCGCCCTGTCGCGAGCTGATCAAGCAATTTCTTCGTTCAATA

Unigene23909 ------------------------------------------------------------
Unigene23910 CGTTGAATAACACGGTTGATCAGCTGGCAACAGTAGCTGCTCAAGCTAATAATACAGCAAACCAGGCTCTTACTTTATCG

Unigene23909 ------------------------------------------------------------
Unigene23910 AATCAAATTTTTGCAAAAGAGAATGATTTGATACTCGGTTGGGGCATATATAATGGTATTAATACATTGTTCCAGATGCG

Unigene23909 ------------------------------------------------------------
Unigene23910 ATCTAATCGGACCACGAACAACATTATGATGACGAATGTGCGTTATAGAGCATCCGTTCGGCTTGCCAACGGCGGCGTAT

Unigene23909 ------------------------------------------------------------
Unigene23910 TACCTTATACTGAATTTTGGATTCGTGGTCCTGGTGAGTTCGGTCAATCGACCTATCCACCGGCACGAGTCCGAACTGGA

Unigene23909 ------------------------------------------------------------
Unigene23910 CAATGGGTTTTGTATTTAACTGGGGGCTCTGGTAGTGCTCAACAATTATTTATAAACCCGTTTAATATACGCGATTATCG

Unigene23909 ------------------------------------------------------------
Unigene23910 ACCATGAATTCCACTAAGGATAAGAAAAAATCGAGCAAACCTGGCTTACGTCAGAGTAACTCGCAACAGCTGCCTCAAAC

Unigene23909 ------------------------------------------------------------
Unigene23910 GGCTATTACAGTTTATAACATGCCCGGTCAAAACCGAGTATTCTTACCAACTGTGCCTCGTACCCCAGAAGATGACGATT

Unigene23909 ------------------------------------------------------------
Unigene23910 CAATTGAAAGTGGTAGTACGGTATCTGAAGTCGGTTTCAGTGAAATTGTGCGGTTGAAGTGTCTAGGACACACAATCGCC

Unigene23909 ------------------------------------------------------------
Unigene23910 GCATATAACCTGCGCTCACGTCGTAAGGGACGTAGTTTACCTATGTATATTCGTGGCGGATGTAGCATGGGTGAAACATT

Unigene23909 ------------------------------------------------------------
Unigene23910 TTCCCAAATGATACCTATTATAGTTAGCCTTCCAACGGTCAATAATGTCATTACAATAGACCCGGTTCTTCCACGTCGTA

Unigene23909 ------------------------------------------------------------
Unigene23910 TGTTAGACTTTGTAACTGCATCCAGCGGTTATGAGTTTATTCGTGCTCAAGTAGTGTGGGTAGTACATATTCCTTCTCCT

Unigene23909 ------------------------------------------------------------
Unigene23910 CTTGGGACGGCTTTGATTTTGCGCGCTTGGGCGCCGGAGTTGGACGCCACGACTGAGACGAGAGGTGTGCGGTGGAAGCC

Unigene23909 ------------------------------------------------------------
Unigene23910 GCAATCTAATACTGCTATCGCATTTAAAATGGACTGGAGCTCTGACATCCCATTTGTCCGTAACACACAGACCTTAACAG

Unigene23909 ------------------------------------------------------------
Unigene23910 CTGTGCGTGACGGACAAAGTGGTTTGTCGCTCAAAATTCAGTGTGTTGAAGACAATTCGACTGAAGCTGTAAACACACCA

Unigene23909 ------------------------------------------------------------
Unigene23910 TTAACTGCAACAGTTTGGTGTTGTGTTTACAACGTCACTATGTCAGGTCAACGCAATTTTACCGAAGCAGATAGAGGAGC

Unigene23909 ------------------------------------------------------------
Unigene23910 TCTTTTAGCTCTTAATTTTAAACCACAATCAACACCATGACAGACACATCAGGAGAAATTCAAGCTGAGGGAGTAAGTAA

Unigene23909 ------------------------------------------------------------
Unigene23910 CCTTACGGTTACTGCCCCTGCCAATACAGATACTACTCCGTTGGTACCTGAGTTAAACCAATCAGCTCCGGCGTCGAAGA

Unigene23909 ------------------------------------------------------------
Unigene23910 AACCCGATTCCTATGCAGGTAAAACAAAGAAGAACCAAATTGGTGCGCTTAACCAGCGTTTCCAACATTTTAATAAGTTC

Unigene23909 ------------------------------------------------------------
Unigene23910 ACTGTAGGTGGTGCCCCCACTTTAACTTGGACTAATATTAATATTAACCCTTATAACATCACAGGCAAAGGTGAGGCGTT

Unigene23909 ------------------------------------------------------------
Unigene23910 TAATCTACCTTTTAGAAGAAACGTTTGGACCAGTGGAAGTATGTCTATGGGTTATTTGTCTACCCTGCAAGTACAAGTTC

Unigene23909 ------------------------------------------------------------
Unigene23910 ATGTAGCTCGACCTCCTCAAGTGTCGGGTACTATACAATTCCGAGATGGAAACAATCCATTGGCAACAATGTATTGTGTC
```

```
Unigene23909 --------------------------------------------------------------------------------
Unigene23910 GATTTCGGTGGTCGTTTGGATTTTCCGTTAGTACCGAACGTTATTAACATCCCGGTGAGACCCCGGCACTGGAATAGTCC

Unigene23909 --------------------------------------------------------------------------------
Unigene23910 ATGGTTCAGAACAGATGAGGCAACTTGTTCTCTGAGTTATAGGCTTATTGCTTTTAATCGTACTGCTGATATCGCTGATG

Unigene23909 --------------------------------------------------------------------------------
Unigene23910 TCACTGTTGACATTTACGTACGCCCTGGCGCGTCGGTGTTTAATACACCGATCAAACCAAAACCTCGCGCAGTTAGTGCT

Unigene23909 --------------------------------------------------------------------------------
Unigene23910 TTGTCTGAGCTCGCTCAGGCTTATCACGACTATGAAGAGGTGGAGTATGCAAAAGTTTACGGTGGTATCATCGAGATCGA

Unigene23909 --------------------------------------------------------------------------------
Unigene23910 GCAGCATGGTATGCCGGAAGAGTTTAGTGACCGCAGTCGCAAAATTCATTTTGACAACTGGTGTGCAGACAACTACGACA

Unigene23909 --------------------------------------------------------------------------------
Unigene23910 TCCACCAGGGTGATGTAGATTTAGCAGAAGCTGAATTTACACCAGTCTGGGATAAGATGTGTGATGATCTAGACGGCAGT

Unigene23909 --------------------------------------------------------------------------------
Unigene23910 GAAGATCACGTCGCCCCCCCAGCTATTGACAGTCCCGAAGAAGGTATGGTTAATTATGTCGAACACCAGGACGACGATAT

Unigene23909 --------------------------------------------------------------------------------
Unigene23910 TGACCAAGACGACTATACAATCCGCGTCTGGGAAGGTGAATTAACTGTAGGTCAACCTATAGCAATCCCTCTCAACCTCT

Unigene23909 --------------------------------------------------------------------------------
Unigene23910 CTGTTCTCAGAGACGTGTCGACGATATCGGATGAAACAACAATCGGACAGAAATTTGAGCGGTTTGCTCATATTATGCCT

Unigene23909 --------------------------------------------------------------------------------
Unigene23910 GCTACTGGAGGTAATCTCGGACCTGAAATTGGCACATATACGATTCATACTCGTTTGCCCACGAATGTTGCAGCATCCAT

Unigene23909 --------------------------------------------------------------------------------
Unigene23910 TGCACATGTATGCGTACCAGATGATCTATCGGACGAAGTTGCCGCTAGAATATTTGGACTCGCTAAGGTGCTGGATATTG

Unigene23909 --------------------------------------------------------------------------------
Unigene23910 CGACATCCGCGATCAGTTCCATTGGAGGACCTCTAATATCAGGTGTAGTACAAACTGCCCCAAAATTGATTTCGACAGTG

Unigene23909 --------------------------------------------------------------------------------
Unigene23910 TTGCCTGGCCCCCTTGGGAGCTTGGCTTCAAAAGTAGTTGGTGGAGTCGCTAATGGATTGTTAGGGGGCAAACCACCAGC

Unigene23909 --------------------------------------------------------------------------------
Unigene23910 CCCACAAGACCCACCAGGTGAGTCGAGTACACCTGCCGCGGTAGGAGGTAAAATTCCAATTGCGCGGTTTCTCGAGTTCC

Unigene23909 --------------------------------------------------------------------------------
Unigene23910 TGAAACCAGTGGCAAGTAACCTTATTGCTAATCCTAGCTTCAGTAACCTTTTAGTTGAACTGATTGATAGTTTCGGCGAT

Unigene23909 --------------------------------------------------------------------------------
Unigene23910 CTGGCATCCCGTGCCACACCAACAATCCCCGTTTCGGTCTACGTTCGTATGACCGGTACCACCGATCGCAATGTCTTTAA

Unigene23909 --------------------------------------------------------------------------------
Unigene23910 CCGCACTATTGTCCCCCGTGATAATGTTGCGAATTTGACCTGGATCCCGCGAGATAGGGTGAGCTATCTCTTCGACATGT

Unigene23909 --------------------------------------------------------------------------------
Unigene23910 TCGGAAATCATCCCAACACTTTTGTGGTGGGGACTCACCAAAACCGTTGCTTCAAGCAACTGATGACAGTAGCGCGTCAG

Unigene23909 --------------------------------------------------------------------------------
Unigene23910 CGCACTAATGTGCCCAGCATTAATTTGCAAGAGGTACTCACGACAGAAGTCCCAGAGGACTTAGATCGTCAAATCCAGAG

Unigene23909 --------------------------------------------------------------------------------
Unigene23910 TTTGCTACTCGCCCGTAATTCTACCGGCCTGATGCAAATTCTGATGACCGCAAGGGAAGAAGCTCCTGCTTTAGGCCCCG

Unigene23909 --------------------------------------------------------------------------------
Unigene23910 ACTGAAACATGTTGGGGAGATAACCGTTTCACAGGAGCGGAGTAATGTTAAGTTTTTTGTATTTGTACAGAGTTTTTCAC

Unigene23909 --------------------------------------------------------------------------------
Unigene23910 ACTTCAAAATAGTTATTTATTTCTCCCTTGTGGGGGTTGAAATAGGTATTTTTAAGTGTTTTCTCTTATATCTAGTGTTT

Unigene23909 --------------------------------------------------------------------------------
Unigene23910 TTTATTATAGATTTACTCCTTCTTTATATTTTATATATGTAGTATATTTATTTTCTATTATTCTATATATTCTTGAAAAC

Unigene23909 -------------
Unigene23910 GTTTGTGAAAAAA
```
