# Supplementary figures and images for: The Novel Agrotis ipsilon Nora Virus Confers Deleterious Effects to the Fitness of Spodoptera frugiperda (Lepidoptera: Noctuidae)
Source: Front Microbiol. 2021 Nov 15;12:727202. doi: 10.3389/fmicb.2021.727202 (PMC8634655; doi:10.3389/fmicb.2021.727202)

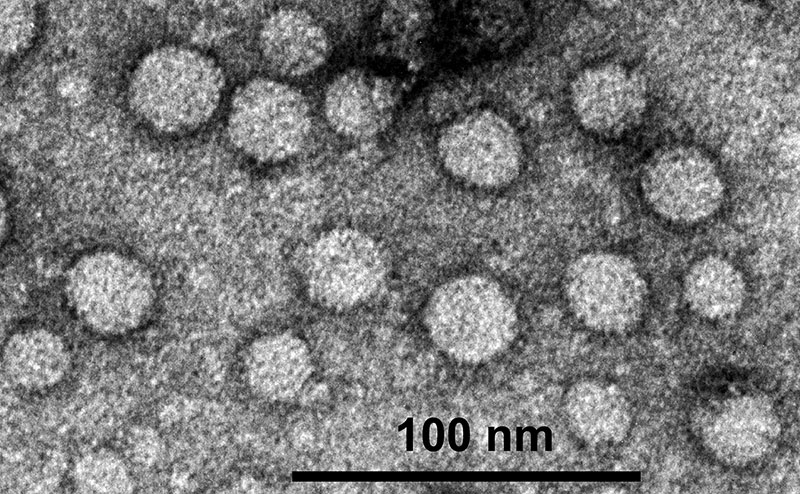

Supplement: Supplementary Figure S3 — Electron microscopy of purified AINV particles. [file Image_1.JPEG]
