# Supplementary material for: The Novel Agrotis ipsilon Nora Virus Confers Deleterious Effects to the Fitness of Spodoptera frugiperda (Lepidoptera: Noctuidae)
Source: Front Microbiol. 2021 Nov 15;12:727202. doi: 10.3389/fmicb.2021.727202 (PMC8634655; doi:10.3389/fmicb.2021.727202)
Supplement: Supplementary Figure S2 — Maximum likelihood phylogeny of Picornavirales and Caliciviridae with RdRp protein sequences. The Nora virus clade was highlighted. Numbers near nodes indicate the ultrafast bootstrap values. The bar indicates the estimated number of substitutions per site. [file Data_Sheet_2.PDF]

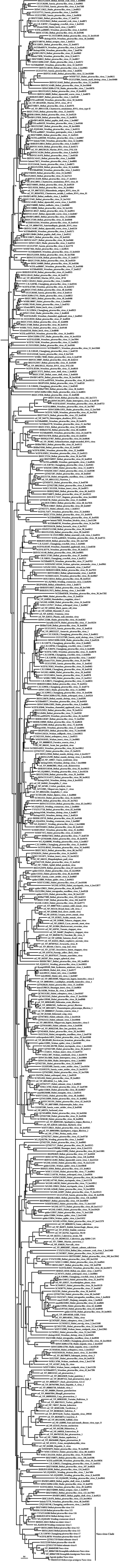
BHZY60932 Beihai picorna-like virus 114
BHHK131520 Beihai picorna-like virus 115
YYSZX16386 Beihai picorna-like virus 116
BHBJDX18741 Beihai shrimp virus 2
WLJQ101491 Wenling crustacean virus 6
BHHZL10411 Beihai sea slater virus 2
WLJQ103138 Wenling crustacean virus 5
BHXG26692 Beihai picorna-like virus 112
BHXG26415 Beihai picorna-like virus 113
CJLX30705 Changjiang picorna-like virus 17
WZFSL80701 Wenzhou picorna-like virus 47
QTM11719 Hubei odonate virus 6
SSZZ2530 Hubei picorna-like virus 66
QTM11719 Hubei odonate virus 6
YP_004849308 Nora virus
YP_009047186 Drosophila subobscura Nora virus
YP_009047190 Drosophila immigrans Nora virus
Agrotis ipsilon Nora virus
MK033133 Helicoverpa armigera Nora virus
Nora virus Clade
0.4
